# Family aggregation of sleep characteristics: Results of the Heinz Nixdorf Recall and the Multi-Generation Study

**DOI:** 10.1371/journal.pone.0252828

**Published:** 2021-06-04

**Authors:** Bernd Kowall, Anna-Therese Lehnich, Sara Schramm, Börge Schmidt, Raimund Erbel, Karl-Heinz Jöckel, Andreas Stang

**Affiliations:** 1 Institute for Medical Informatics, Biometry and Epidemiology, Medical Faculty, University Duisburg-Essen, Essen, Germany; 2 School of Public Health, Department of Epidemiology Boston University, Boston, MA, United States of America; Sapienza University of Rome, ITALY

## Abstract

**Introduction:**

Poor sleep is a risk factor for adverse health events. For health prevention, it may be helpful to know whether poor sleep or sleep disorders in individuals are associated with sleep problems in their partners or children.

**Methods:**

In the MultiGeneration Study (MGS, conducted from 2013 to 2016), 1237 partners (aged 27 to 90 years) and 1660 adult children (aged 18 to 66 years) of index persons were recruited. Index persons are participants of the Heinz Nixdorf Recall Study, a population-based cohort study in the Ruhr area (study start 1999–2001, 4841 participants aged 45–75 years). We used two analysis populations: one with 1181 index persons whose partners were in MGS, and one with 1083 index persons with at least one adult child in MGS. Sleep characteristics were assessed using questionnaires (including the Pittsburgh Sleep Quality Index). The exposure was the presence of a sleep characteristic of the index subject.

**Results:**

Children showed the investigated sleep characteristics more often if these were also present in their parent (e.g., RR (relative risk) = 1.28 (95% CI: 1.06–1.55) for poor sleep quality). In partners, strong associations were observed for rising times and napping, but only weak associations for snoring, poor sleep quality and sleep disorders. Snoring of the bed partner is a risk factor for poor sleep (e.g., RR = 1.67 (0.91–3.07) for difficulties falling asleep).

**Conclusion:**

Aggregation is observed for many sleep characteristics in people living in partnerships as well as in parents and their adult children.

## Introduction

Sleep characteristics are associated with health outcomes. For instance, a U-shaped association between sleep duration and coronary heart disease and stroke, respectively, has been reported [1, 2]. Moreover, there is evidence for an association between sleep duration and hypertension [3–7]. Short and long sleep duration, and sleep disorders are associated with a higher risk of diabetes [8–11]. In addition, short and long nocturnal sleep duration and daytime napping are associated with increased all-cause mortality [12]. Moreover, obstructive sleep apnea (OSA) is associated with severe health outcomes: OSA patients have a higher risk of olfactory dysfunction, they have more oxidative stress, higher levels of inflammation biomarkers, and a higher risk of cardiovascular disease [13, 14].

Seeing this impact of sleep on health, measures to improve poor sleep are warranted. Sleep hygiene includes various recommendations for better sleep although there is no full consensus on the list of such recommendations [15]. Patients may profit from better sleep hygiene although sleep hygiene is not a sufficient therapy for insomnia [16]. Furthermore, “one shot” interventions which include a short treatment session may be helpful to avoid chronification of acute insomnia [16].

If a person suffers from sleep disorders, it is of interest whether sleep disorders are also present in relatives or partners so that both could profit from measures of sleep improvement. So far, there are only few studies on familial insomnia [17–21], and even less studies on aggregation of sleep characteristics in couples [21].

The present study aims to examine whether sleep characteristics and sleep habits are more prevalent in partners and offspring, respectively, given that an index person displays these sleep characteristics. Moreover, we investigate whether persons suffer more often from poor sleep and sleep disorders when their partners snore.

## Materials and methods

The study was approved by the Ethical Committee of the Medical Faculty of the University Clinic Essen. All participants gave their written informed consent.

### Study population

The ongoing prospective population-based Heinz Nixdorf Recall (HNR) cohort study is carried out in three large adjacent German cities (Mülheim, Essen, Bochum) in the Ruhr district. Details of the aims and the design of the study have been published earlier [22]. Briefly, participants were invited to the study center for the first time between 2000 and 2003 (T0), and 4814 persons (mean age 59.6 years, age range between 45 and 75 years) participated in the baseline examinations. The next visits to the study center took place between 2006 and 2008 (T1) with 4157 participants (mean age 64.4 years, age range 50 to 80 years) and between 2011 and 2015 (T2) with 3089 participants (mean age 68.7 years, age range 55 to 86 years). The median time between T0 and T1 was 5.1 years, and 5.2 years between T1 and T2. During the examination in the study center, participants filled in questionnaires, took part in face-to-face interviews and underwent physical examinations including extensive laboratory tests. Additionally, participants received annual postal questionnaires.

**Fig 1:**
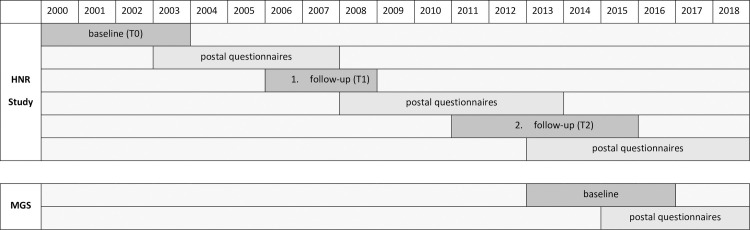
Study design of the Heinz Nixdorf Recall (HNR) Study and Heinz Nixdorf Recall MultiGenerationStudy (MGS).

The Heinz Nixdorf Recall MultiGenerationStudy (MGS) comprises partners and children of HNR participants in order to investigate cardiovascular risk factor burden and the related incidence of cardiovascular disease within families. Briefly, from 2013 to 2016, 1237 partners and 1660 adult children of HNR participants, aged 18 to 90 years, underwent a similar examination program as participants of the HNR Study at T2, and completed annual postal questionnaires on health status since then. Fig 1 shows the study design of the HNR Study and MGS. We used data from the third examination (T2) of the HNR Study whose participants served as index persons and data from the MGS.

Of the 1237 partners in MGS, 1181 had an index person who was examined at T2. Thus, analysis population A included 1181 index persons and their partners. Of the 1660 children in MGS, 1607 were biological children, and of those, 1497 had an index person who took part at T2. 735 index persons had one child in MGS, another 289 index persons had two children in MGS, 52 index persons had three children in MGS, and 7 index persons had four children in MGS. Thus, analysis population B included 1083 index persons and 1497 children.

### Assessment of sleep characteristics

Sleep characteristics taken into account in the present study were assessed in an identical way for index persons and their partners / children.

Sleep quality was assessed using an item from the Pittsburgh Sleep Quality Index (PSQI): “How would you rate the overall quality of your sleep during the last four weeks?” (very good / good / poor / very poor) [23]. Sleep disorders were assessed using two other items from the PSQI: „During the last four weeks, how often have you had trouble sleeping because you could not fall asleep within 30 minutes?“, and „During the last four weeks, how often have you slept poorly because you woke up in the middle of the night or early in the morning?”(Not at all during the last four weeks / less than once a week / once or twice per week / three times or more per week). Preferences for rising times were assessed from the following item: „If it were only up to your own well-being and you could plan your day completely freely, when would you get up?”(mark only one hour). Snoring was assessed using the item „Do you snore?”(yes / no). An item on napping was „How frequently do you usually nap?”(never / less than once a week / one to four times per week / five to six times per week / daily).

### Statistical analyses

Log-binomial regression models were fitted to estimate relative risks (RRs) with 95% confidence intervals (CI) for associations between sleep characteristics of the index persons as the exposure and sleep characteristics of their partners and children, respectively, as the outcome. We also fitted log-binomial models to estimate RRs (95% CI) for the association between snoring of the index person and sleep characteristics of the partner. To analyze associations between sleep characteristics of the index persons and sleep characteristics of their children, another approach was additionally used to take into account that data of siblings may be correlated: We fitted logistic regression models with random effects to estimate odds ratios (ORs) with 95% CI.

For the regression analyses, sleep characteristics were transformed into binary variables as follows: very poor / poor versus very good / good for sleep quality; three times or more per week versus less often for sleep disorders; once a week or more versus less often for napping; yes versus no for snoring. Persons who preferred getting up before 7 o´clock a.m. were defined as early risers, and those who preferred getting up at 9 o´clock a.m. or later as late risers.

We did several sensitivity analyses:

First, in the analysis of study population A, couples who reported not to sleep in the same room were excluded when the sleep characteristics were snoring, sleep quality or sleep disorders. Second, analyses on the associations between sleep characteristics of index persons and their children were stratified by the median of parental age. Third, although our aim was to analyze associations and not causal inference, we added some adjusted analyses on the association between sleep characteristics of index persons and their children (adjustment for age and sex; adjustment for age, sex, BMI, smoking, chronic diseases and drug intake).

All statistical analyses were performed using SAS version 9.4.

## Results

The mean age of the index persons was 67.2 years in analysis population A, and 69.0 years in analysis population B (Tables 1 and 2). The mean age of the children of the index persons was 42.6 years. In both analysis populations, about 80% of the index persons reported good or very good sleep quality. In analysis population B, adult children more often reported poor or very poor sleep than their parents (28% versus 18%). In analysis population A, 21% of the index persons reported difficulties maintaining sleep or early morning awakening at least three times a week, 11% reported difficulties falling asleep at least three times a week (analysis population B: 22% and 14%, respectively). 43% of the index persons used to nap at least once a week in both analysis populations. 49 and 41%, respectively, gave a self-report of snoring in analysis population A and B. However, 24% and 30% did not know whether they snored, or did not answer this question. In both study populations, 5% of the index persons had a preference for getting up before 7 o´clock a.m., and 21% and 20%, respectively, preferred getting up at 9 o´clock a.m. or later. The proportions of persons with chronic diseases like diabetes mellitus, cancer, stroke, and coronary heart disease were similar in index persons and their partners (S1 Table). Likewise, the proportions of persons taking antihypertensive medication or cholesterol lowering drugs were similar in index persons and their partners. Adult children had chronic diseases or took drugs considerably less often than their parents (e.g., coronary heart disease 9.2% in the index persons, and 0.9% in their children) (S2 Table).

**Table 1:** Characteristics of index persons and their partners: The Heinz Nixdorf Recall (HNR) and the MultiGenerationStudy (MGS) (analysis population A).

		Index person (HNR T2)	Partners (MGS)
N		1181	1181
Age (years)		67.2 ± 6.6	67.8 ± 7.7
Sex (male) (%) ^a^		672 (56.9%)	512 (43.3%)
Sleep quality	poor / very poor	193 (16.3%)	260 (22.0%)
	good / very good	961 (81.4%)	886 (75.0%)
	Missing	27 (2.3%)	35 (3.0%)
Snoring	Yes	582 (49.3%)	500 (42.3%)
	No	311 (26.3%)	395 (33.4%)
	Don´t know / missing	288 (24.4%)	286 (24.2%)
Napping	≥ 1 time / week	512 (43.4%)	490 (41.5%)
	< 1 time / week	619 (52.4%)	638 (54.0%)
	Don´t know / missing	50 (4.2%)	53 (4.5%)
Difficulties falling asleep ^b^	≥ 3 times / week	128 (10.8%)	96 (8.1%)
	< 3 times / week	1013 (85.8%)	863 (73.1%)
	Don´t know / missing	40 (3.4%)	222 (18.8%)
Difficulties maintaining sleep / early morning awakening ^b^	≥ 3 times / week	242 (20.5%)	173 (14.6%)
	< 3 times / week	897 (76.0%)	780 (66.0%)
	Don´t know / missing	42 (3.6%)	228 (19.3%)
Preference for getting up early ^b^^,^^c^	Yes	61 (5.2%)	65 (5.5%)
	No	1,097 (92.9%)	890 (75.3%)
	Missing	23 (1.9%)	226 (19.1%)
Preference for getting up late ^b^^,^^d^	Yes	247 (20.9%)	180 (15.2%)
	No	911 (77.1%)	775 (65.6%)
	Missing	23 (1.9%)	226 (19.1%)
Sleeping in the same room with partner	Yes	959 (81.2%)	-
	No	179 (15.2%)	
	Missing	43 (3.6%)	

Mean ± standard deviation; n (proportions (%)).

HNR T2: third visit to the study center in the Heinz Nixdorf Recall Study; MGS: MultiGeneration Study.

^a^ Three male index persons have male partners, therefore, the sum of male index persons and male partners is 1,184.

^b^ Data for partners were based on self-administered questionnaires mailed after MGS baseline.

^c^ Before 7 o´clock a.m.

^d^ At 9 o´clock a.m. or later.

**Table 2:** Characteristics of index persons and their children: the Heinz Nixdorf Recall (HNR) and the MultiGenerationStudy (MGS) (analysis population B).

		Index person per family (HNR T2)	Index person per child (HNR T2)	Children (MGS)
N		1083	1497	1497
Number of children in MGS per index person	1	735	-	-
	2	289	-	-
	3	52	-	-
	4	7	-	-
Age (years)		69.0 ± 7.3	69.1 ± 7.4	42.6 ± 9.5
Sex (male) (%)		486 (44.9%)	687 (45.9%)	684 (45.7%)
Sleep quality	poor / very poor	197 (18.2%)	276 (18.4%)	415 (27.7%)
	good / very good	859 (79.3%)	1181 (78.9%)	1031 (68.9%)
	missing	27 (2.5%)	40 (2.7%)	51 (3.4%)
Snoring	Yes	440 (40.6%)	610 (40.7%)	474 (31.7%)
	No	319 (29.5%)	436 (29.1%)	634 (42.4%)
	Don´t know / missing	324 (29.9%)	451 (30.1%)	389 (26.0%)
Napping	≥ 1 time / week	461 (42.6%)	637 (42.6%)	245 (16.4%)
	< 1 time / week	575 (53.1%)	794 (53.0%)	1187 (79.3%)
	Don´t know / missing	47 (4.3%)	66 (4.4%)	65 (4.3%)
Difficulties falling asleep ^a^	≥ 3 times / week	146 (13.5%)	210 (14.0%)	97 (6.5%)
	< 3 times / week	900 (83.1%)	1233 (82.4%)	1009 (67.4%)
	Don´t know / missing	37 (3.4%)	54 (3.6%)	391 (26.1%)
Difficulties maintaining sleep / early morning awakening ^a^	≥ 3 times / week	236 (21.8%)	334 (22.3%)	216 (14.4%)
	< 3 times / week	815 (75.3%)	1113 (74.3%)	885 (59.1%)
	Don´t know / missing	32 (2.9%)	50 (3.3%)	396 (26.5%)
Preference for getting up early ^a^^,^^b^	Yes	58 (5.4%)	83 (5.5%)	56 (3.7%)
	No	1000 (92.3%)	1378 (92.1%)	1048 (70.0%)
	Missing	25 (2.3%)	36 (2.4%)	393 (26.3%)
Preference for getting up late ^a^^,^^c^	Yes	220 (20.3%)	301 (20.1%)	333 (22.2%)
	No	838 (77.4%)	1160 (77.5%)	771 (51.5%)
	Missing	25 (2.3%)	36 (2.4%)	393 (26.3%)

Mean ± standard deviation; n (proportions (%)).

HNR T2: third visit to the study center in the Heinz Nixdorf Recall Study; MGS: MultiGeneration Study.

^a^ For the children of the index persons, data were based on self-administered questionnaires mailed to participants after MGS baseline.

^b^ Before 7 o´clock a.m.

^c^ At 9 o´clock a.m. or later.

**Fig 2:**
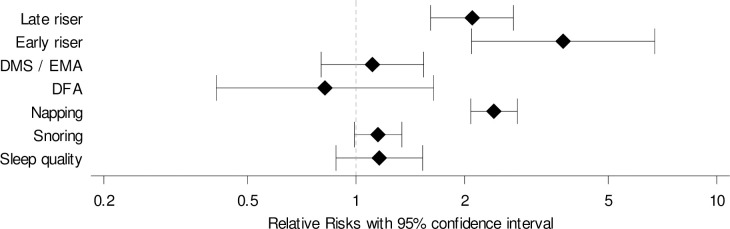
Relative risks (95% CI) for associations between sleep characteristics of index persons and their partners. Late riser: Person who prefers getting up at 9 o´clock a.m. or later. Early riser: Person who prefers getting up before 7 o´clock a.m. DMS / EMA: difficulties maintaining sleep / early morning awakening. DFA: difficulties falling asleep.

Persons with a partner who used to nap were 2.4 (95% CI: 2.1–2.8) times more likely to nap also than persons with a partner who did not nap (Table 3, Fig 2). When the index person preferred getting up early or late, the partner had an increased probability for the same preference (RR = 3.8 (2.1–6.7), and RR = 2.1 (1.6–2.7), respectively). For sleep quality, snoring, and sleep disorders, associations between sleep characteristics of the index persons and their partners were small. Relative risks changed only slightly after exclusion of couples who did not sleep in the same room (RR = 1.19 (0.87–1.62) for poor sleep quality, RR = 1.17 (0.99–1.38) for snoring, 0.80 (0.36–1.79) for difficulties falling asleep, and 0.97 (0.64–1.46) for difficulties maintaining sleep / early morning awakening).

**Table 3:** Associations between sleep characteristics of index persons and their partners, results from log-binomial regression analyses.

Sleep characteristics of index persons	n	Sleep outcome of partners ^a^	RR (95% CI)
Sleep quality			
poor / very poor	183	46 (25.1%)	1.16 (0.88–1.53)
good / very good	940	204 (21.7%)	1
Snoring			
Yes	438	243 (55.5%)	1.11 (0.95–1.28)
No (ref)	247	124 (50.2%)	1
Napping			
≥ 1 time / week	492	318 (64.6%)	2.41 (2.08–2.80)
< 1 time / week (ref)	590	158 (26.8%)	1
Difficulties falling asleep			
≥ 3 times / week	96	8 (8.3%)	0.82 (0.41–1.64)
< 3 times / week	835	85 (10.2%)	1
Difficulties maintaining sleep / early morning awakening			
≥ 3 times / week	182	36 (19.8%)	1.11 (0.80–1.54)
< 3 times / week	739	132 (17.9%)	1
Preference for getting up early ^b^			
Yes	50	11 (22.0%)	3.75 (2.09–6.73)
No	887	52 (5.9%)	1
Preference for getting up late ^c^			
Yes	203	65 (32.0%)	2.10 (1.61–2.73)
No	734	112 (15.3%)	1

RR: relative risk; CI: confidence interval.

^a^ For sleep quality of the index person as the exposure, the corresponding outcome of the partners is poor / very poor sleep quality; for snoring (yes / no) of the index person as the exposure, the corresponding outcome of the children is snoring (yes), etc.

^b^ Before 7 o´clock a.m.

^c^ At 9 o´clock a.m. or later.

**Fig 3:**
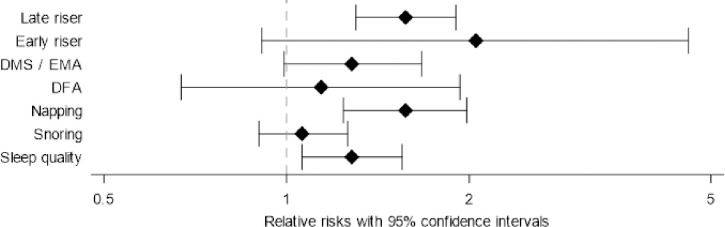
Relative risks (95% CI) for associations between sleep characteristics of index persons and their children. Late riser: Person who prefers getting up at 9 o´clock a.m. or later. Early riser: Person who prefers getting up before 7 o´clock a.m. DMS/EMA: difficulties maintaining sleep / early morning awakening. DFA: difficulties falling asleep.

If the index persons reported poor / very poor sleep quality, their children were 1.3 (1.1–1.6) times more likely to assess their sleep quality as poor / very poor than children whose parents reported good / very good sleep quality (Table 4, Fig 3). Snoring of the index persons was barely associated with snoring of the children (RR = 1.1 (0.9–1.3)). Children of index persons who used to nap were more likely to nap also (RR = 1.6 (1.2–2.0)). Children of index persons who had difficulties maintaining sleep or early morning awakening were more likely to have difficulties maintaining sleep or early morning awakening (RR = 1.3 (1.0–1.7)). Children of late risers were more likely to prefer getting up late (RR = 1.6 (1.3–1.9)). When the analyses on index persons and their children were stratified by the median of parental age, for sleep quality, snoring, napping and preference for getting up early relative risks were higher for parental age ≤ 69 years (e.g., for sleep quality, RR = 1.48 (95% CI: 1.15–1.91) for parental age ≤ 69 years, and RR = 1.10 (95% CI: 0.82–1.46) for parental age > 69 years) (S3 Table). Associations between sleep characteristics of index persons and their children were only slightly changed after adjustment for age, sex, BMI, smoking, chronic diseases (cancer, diabetes, stroke, coronary heart disease) and drug intake (antihypertensive drugs, cholesterol lowering drugs, benzodiazepines) (S4 Table).

**Table 4:** Associations between sleep characteristics of index persons and their children.

Sleep characteristics of index persons	n	Sleep outcome of children ^a^	RR (95% CI) ^b^	OR (95% CI) ^b^
Sleep quality				
poor / very poor	264	92 (34.9%)	1.28 (1.06–1.55)	1.43 (1.07–1.91)
good / very good	1145	312 (27.3%)	1	1
Snoring				
Yes	462	197 (42.6%)	1.06 (0.90–1.26)	1.11 (0.82–1.50)
No (ref)	327	131 (40.1%)	1	1
Napping				
≥ 1 time / week	606	131 (21.6%)	1.57 (1.24–1.98)	1.72 (1.29–2.31)
< 1 time / week (ref)	762	105 (13.8%)	1	1
Difficulties falling asleep				
≥ 3 times / week	155	15 (9.7%)	1.14 (0.67–1.93)	1.15 (0.64–2.07)
< 3 times / week	918	78 (8.5%)	1	1
Difficulties maintaining sleep / early morning awakening				
≥ 3 times / week	251	60 (23.9%)	1.28 (0.99–1.67)	1.38 (0.97–1.95)
< 3 times / week	818	152 (18.6%)	1	1
Preference for getting up early ^c^				
Yes	61	6 (9.8%)	2.05 (0.91–4.59)	2.17 (0.87–5.42)
No	1019	49 (4.8%)	1	1
Preference for getting up late ^d^				
Yes	224	95 (42.4%)	1.57 (1.30–1.90)	2.00 (1.45–2.76)
No	856	231 (27.0%)	1	1

RR: relative risk; OR: odds ratio; CI: confidence interval.

^a^ For sleep quality of the index person as the exposure, the corresponding outcome of the children is poor / very poor sleep quality; for snoring (yes / no) of the index person as the exposure, the corresponding outcome of the children is snoring (yes), etc.

^b^ Relative risks were estimated from log-binomial models, odds ratios were estimated from logistic regression models with random effects.

^c^ Before 7 o´clock a.m.

^d^ At 9 o´clock a.m. or later.

Partners of index persons who snored had increased risks of sleep problems (Table 5). If the index persons snored, their partners were 1.7 (0.9–3.1) times more likely to report difficulties falling asleep. For difficulties maintaining sleep or early morning awakening, the corresponding relative risk was 1.9 (1.2–3.0). Partners of index persons who snored were 1.4 (1.0–1.9) times more likely to report poor sleep quality than partners of non-snorers.

**Table 5:** Associations between snoring of the index persons and sleep characteristics of their partners, results from log-binomial models.

Sleep characteristic partner		Snoring of the index person		RR (95% CI)
		yes	no	
Difficulties falling asleep	yes	37 (9.8%)	13 (5.9%)	1.67 (0.91–3.07)
	no	341 (90.2%)	209 (94.1%)	1
Difficulties maintaining sleep / early morning awakening	yes	72 (19.1%)	22 (10.0%)	1.91 (1.22–2.95)
	no	306 (80.9%)	199 (90.0%)	1
Poor sleep quality	yes	116 (25.0%)	47 (18.1%)	1.38 (1.02–1.87)
	no	348 (75.0%)	213 (81.9%)	1

RR: relative risk; CI: confidence interval.

## Discussion

We found evidence that adult children more often report napping or poor sleep quality when their parents report napping or poor sleep. Couples share preferences for rising times or napping, but sleep disorders in one partner are barely associated with sleep disorders in the other. Having a snoring partner is associated with poor sleep quality and sleep disorders.

### Comparison with earlier studies

In the present study, poor sleep was more frequent in the adult children than in their parents. This is not consistent with earlier results showing that sleep latency increases with age while sleep maintenance and deep sleep decreases [24–26]. However, the offspring belong to another age cohort, and little is known about trends in the prevalence of sleep problems and insomnia in Germany [27]. In addition, employment status may have an impact on sleep quality, and the adult children of the MGS were mostly employed in contrast to their parents.

In two clinical studies, familial aggregation of insomnia was found [18, 19]. In one study which lacked a control group, 35% of insomnia patients reported that at least one family member had insomnia also; in the other study, familial insomnia was reported most often by patients with primary insomnia, less often by patients with psychiatric insomnia, and least often by control persons without insomnia (73%, 43%, and 24%, respectively). Contrary to the present study, the relatives were not interviewed in these clinical studies. This also applies to a population-based cross-sectional study which showed that persons who ever suffered from insomnia had an increased chance of having relatives with insomnia compared to persons without a history of insomnia (OR = 1.57, 95% CI: 1.19–2.07) [20]. In a further study with a reconstructed cohort design, biological relatives of index persons had a higher risk of insomnia when index persons were affected with insomnia but results were not unambiguous for parents and children (RR = 1.80 (p = 0.04) for all biological relatives, 4.96 (p = 0.08) for siblings, 0.84 (p = 0.71) for parents, 1.65 (p = 0.38) for offspring) [21]. In the same study, partners of index persons were also taken into account, and the corresponding relative risk was increased but not estimated precisely (RR = 2.13, p = 0.28). In line with the present study, Gunn et al. found that into-bed times were positively correlated within couples [28].

Contrary to earlier studies, we also did age-stratified analyses on the associations between sleep characteristics of index persons and their children. For sleep quality, napping and snoring, these associations were stronger for parents younger than the median parental age of 69 years than for older parents. This is plausible because these associations may partly result from shared life styles or shared environments and because such factors may match less the longer parents and children live apart.

Our finding that partners of snorers are more often affected from sleep disorders and poor sleep quality is in accordance with earlier results from a study in Sweden, which showed that women of heavy snorers more often suffer from insomnia and daytime sleepiness than women whose partners do not snore [29]. In a recent study on snorers, it was reported that sound levels of snorers exceeded 45 dB(A) in two thirds of the participants so that the authors considered snoring as a “noise pollution in the bedroom” [30]. Thus, there is evidence that snoring bedpartners are a risk factor for poor sleep.

We did not adjust for potential confounders because we were interested in associations and not in causal inferences. For associational questions, a mere comparison is sufficient [31]. For example, we wanted to know whether partners of index persons were more likely to have sleep disorders if the index person was affected. We did not want to investigate whether difficulties falling asleep in the index persons cause this sleep disorder in the partners. To answer the latter question, potential confounders like the noise level in the bedroom or shared problems which cause stress in both partners would have to be taken into account. Additionally, we adjusted the analysis on sleep characteristics of parents and children for age, sex, BMI, smoking, chronic diseases and drug intake. This adjustment had only little impact on the strength of the associations. This seems plausible as the proportion of individuals taking drugs or having chronic diseases was much lower in the adult children than in their parents. Thus, the observed associations do not result from confounding paths where, e.g., drug intake of the index person causes sleep problems in the index person on the one hand, and causes drug intake in the children on the other hand which in turn causes sleep problems in the child.

### Public health relevance

Poor sleep is a risk factor for adverse health events [1–12]. Our study confirms that adult children are more likely to suffer from sleep disorders and poor sleep quality when their parents are affected. As past studies have shown familial aggregation also in other sleep related diseases like obstructive sleep apnea or restless leg syndrome [32, 33], physicians should generally give consideration to first-degree relatives of patients who are affected with sleep disorders.

Snoring is strongly associated with obstructive sleep apnea which has a high incidence in the older population [34]. Our findings on the effects of snoring on bed partners’ sleep is a second reason why snoring should be given consideration. An earlier study suggested that bed partners of snorers may benefit from treating snorers: continuous positive airways pressure (CPAP) was used to eliminate snoring, and bed partner’s sleep quality improved strongly [35].

### Limitations and strengths

In this study, sleep characteristics were assessed by self-report. This is not a limitation with regard to preferences for rising times or frequency of napping. However, in an earlier study, agreement between self-reports and partners’ reports of snoring was rather poor [36], and, thus, self-report of snoring may lead to misclassification. Furthermore, we only assessed sleep disorders during the last four weeks so that we could not distinguish between early and late onset sleep problems. Earlier research suggested that familial aggregation was stronger for early than for late onset of insomnia [17]. Our study also has strengths. It is population-based with fairly large study groups. Contrary to some other studies, sleep characteristics of offspring and partners were directly assessed. Thus, reporting bias could be avoided because in earlier studies there was an indication that persons with insomnia had a tendency to overreport insomnia in relatives [21].

### Conclusions

There are only few studies on familial aggregation of sleep characteristics. We found aggregation of sleep disorders and poor sleep quality in parents and their offspring, but not in couples. Moreover, we showed that bed partners of snorers are more often affected from poor sleep and sleep disorders.

## Supporting information

S1 Table Additional characteristics of index persons and their partners: The Heinz Nixdorf Recall (HNR) and the MultiGenerationStudy (MGS) (analysis population A). (DOCX)

S2 Table Additional characteristics of index persons and their children: The Heinz Nixdorf Recall (HNR) and the MultiGenerationStudy (MGS) (analysis population B). (DOCX)

S3 Table Associations between sleep characteristics of index persons and their children, stratified by the median of parental age. (DOCX)

S4 Table Crude and adjusted associations between sleep characteristics of index persons and their children. (DOCX)

## References

[1] Cappuccio FP, Cooper D, Délia L, Strazzullo P, Miller MA. Sleep duration predicts cardiovascular outcomes: a systematic review and meta-analysis of prospective studies. Eur Heart J. 2011; 32: 1484–1492. doi: 10.1093/eurheartj/ehr007 21300732

[2] Yin J, Jin X, Shan Z et al. Relationship of sleep duration with all-cause mortality and cardiovascular events: A systematic review and dose-response meta-analysis of prospective cohort studies. J Am Heart Assoc. 2017; 6:e005947. doi: 10.1161/JAHA.117.005947 28889101 PMC5634263

[3] Wang Q, Xi B, Liu M, Zhang Y, Fu M. Short sleep duration is associated with hypertension risk among adults: a systematic review and meta-analysis. Hypertens Res. 2012; 35: 1012–1018. doi: 10.1038/hr.2012.91 22763475

[4] Guo X, Zheng L, Wang J et al. Epidemiological evidence for the link between sleep duration and high blood pressure: A systematic review and meta-analysis. Sleep Medicine. 2013; 14: 324–332. doi: 10.1016/j.sleep.2012.12.001 23394772

[5] Meng L, Zheng Y, Hui R. The relationship of sleep duration and insomnia to risk of hypertension incidence: a meta-analysis of prospective cohort studies. Hypertens Res. 2013; 36: 985–995. doi: 10.1038/hr.2013.70 24005775 PMC3819519

[6] Gangwisch JE. A review of evidence for the link between sleep duration and hypertension. Am J Hypertens. 2014; 27: 1232–1242. doi: 10.1093/ajh/hpu148 24778107 PMC4229731

[7] Wang Y, Mei H, Jiang YR et al. Relationship between duration of sleep and hypertension in adults: a meta-analysis. J Clin Sleep Med. 2015; 11: 1047–1056. doi: 10.5664/jcsm.5024 25902823 PMC4543249

[8] Cappuccio FP, D´Elia L, Strazzullo P, Miller MA. Quantity and quality of sleep and incidence of type 2 diabetes. A systematic review and meta-analysis. Diabetes Care. 2010; 33: 414–420 doi: 10.2337/dc09-1124 19910503 PMC2809295

[9] Holliday EG, Magee CA, Kritharides L, Banks E, Attia J. Short sleep duration is associated with risk of future diabetes but not cardiovascular disease: a prospective study and meta-analysis. PLoS ONE. 2013; 8: e82305. doi: 10.1371/journal.pone.0082305 24282622 PMC3840027

[10] Shan Z, Ma H, Xie M et al. Sleep duration and risk of type 2 diabetes: A meta-analysis of prospective studies. Diabetes Care. 2015; 38: 529–537 doi: 10.2337/dc14-2073 25715415

[11] Larcher S, Benhamou PY, Pépin JL, Borel AL. Sleep habits and diabetes. Diabetes Metab. 2015; 41: 263–271. doi: 10.1016/j.diabet.2014.12.004 25623152

[12] Da Silva AA, de Mello RG, Schaan CW, Fuchs FD, Redline S, Fuchs SC. Sleep duration and mortality in the elderly: a systematic review with meta-analysis. BMJ Open. 2016; 6(2):e008119. doi: 10.1136/bmjopen-2015-008119 26888725 PMC4762152

[13] Ianella G, Magliulo G, Maniaci A et al. Olfactory function in patients with obstructive sleep apnea: a meta-analysis study. Eur Arch Otorhinolaryngol 2021; 278: 883–891. doi: 10.1007/s00405-020-06316-w 32914257

[14] Maniaci A, Ianella G, Cocuzza S et al. Oxidative stress and inflammation biomarker expression in obstructive sleep apnea patients. J Clin Med 2021; 10:277. doi: 10.3390/jcm10020277 33451164 PMC7828672

[15] Stepanski EJ, Wyatt JK. Use of sleep hygiene in the treatment of insomnia. Sleep Med Rev. 2003; 7: 215–225. doi: 10.1053/smrv.2001.0246 12927121

[16] Ellis JG, Allen SF. Sleep hygiene and the prevention of chronic insomnia. In: Grandner MA (editor), Sleep and Health. Elsevier; 2019. pp. 137–145.

[17] Dauvilliers Y, Morin CM. Heritability and genetic factors in chronic insomnia. In: Shaw P, Tafti M & Thorpy M (editors). The Genetic Basis and Sleep Disorders. Cambridge University Press; 2013. pp. 227–234.

[18] Bastien CH, Morin CM. Familial incidence of insomnia. J Sleep Res. 2000; 9: 49–54. doi: 10.1046/j.1365-2869.2000.00182.x 10733689

[19] Dauvilliers Y, Morin C, Cervena K et al. Family studies in insomnia. J Psychosom Res. 2005; 58: 271–278. doi: 10.1016/j.jpsychores.2004.08.012 15865952

[20] Beaulieu-Bonneau S, LeBlanc M, Mérette C, Dauvilliers Y, Morin CM. Family history of insomnia in a population-based sample. Sleep. 2007; 30: 1739–1745. doi: 10.1093/sleep/30.12.1739 18246983 PMC2276141

[21] Jarrin DC, Morin CM, Rochefort A et al. Familial aggregation of insomnia. Sleep. 2017; 40(2). doi: 10.1093/sleep/zsw053 28364499

[22] Schmermund A, Möhlenkamp S, Stang A, Grönemeyer D, Seibel R, Hirche H et al. Assessment of clinically silent atherosclerotic disease and established and novel risk factors for predicting myocardial infarction and cardiac death in healthy middle-aged subjects: Rationale and design of the Heinz Nixdorf RECALL Study. Risk factors, evaluation of coronary calcium and lifestyle. Am Heart J. 2002; 144: 212–218. doi: 10.1067/mhj.2002.123579 12177636

[23] Buysse DJ, Reynolds CF, Monk TH, Berman SR, Kupfer DJ. The Pittsburgh sleep quality index: a new instrument for psychiatric practice and research. Psychiatry Res. 1989; 28, 193–213. doi: 10.1016/0165-1781(89)90047-4 2748771

[24] Ohayon MM, Carskadon MA, Guilleminault C, Vitiello MV. Meta-analysis of quantitative sleep parameters from childhood to old age in healthy individuals: developing normative sleep values across the human lifespan. Sleep. 2004; 27: 1255–1273. doi: 10.1093/sleep/27.7.1255 15586779

[25] Vitiello MV. Sleep in normal aging. Sleep Med Clin. 2006; 1: 171–176. 10.1016/j.jsmc.2017.09.001 PMC5841578 29412976

[26] Li J, Vitiello MV, Gooneratne NS. Sleep in normal aging. Sleep Med Clin. 2018; 13: 1–11. doi: 10.1016/j.jsmc.2017.09.001 29412976 PMC5841578

[27] Schlack R, Hapke U, Maske U, Busch MA, Cohrs S. Häufigkeit und Verteilung von Schlafproblemen und Insomnie in der deutschen Erwachsenenbevölkerung. Ergebnisse der Studie zur Gesundheit Erwachsener in Deutschland (DEGS1). Bundesgesundheitsbl. 2013; 56: 740–748. 10.1007/s00103-013-1689-2 23703493

[28] Gunn HE, Buysse DJ, Hasler BP, Begley A, Troxel WM. Sleep concordance in couples is associated with relationship characteristics. Sleep. 2015; 38: 933–939. doi: 10.5665/sleep.4744 25581920 PMC4434560

[29] Ulfberg J, Carter N, Talbäck M, Edling C. Adverse health effects among women living with heavy snorers. Health Care Women Int. 2000; 21: 81–90. doi: 10.1080/073993300245311 10818830

[30] Sowho M, Sgambati F, Guzman M, Schneider H, Schwartz A. Snoring: a source of noise pollution and sleep apnea predictor. Sleep. 2020; 43: zsz305. doi: 10.1093/sleep/zsz305 31837267 PMC8152862

[31] Hernan M. The C-Word: Scientific euphemisms do not improve causal inference from observational data. Am J Public Heath. 2018; 108: 616–619. 10.2105/AJPH.2018.304337 PMC5888052 29565659

[32] Redline S, Tishler PV, Tosteson TD et al. The familial aggregation of obstructive sleep apnea. Am J Respir Crit Care Med. 1995; 151 (3 Pt 1): 682–687. doi: 10.1164/ajrccm/151.3_Pt_1.682 7881656

[33] Parish JM. Genetic and immunologic aspects of sleep and sleep disorders. Chest. 2013; 143: 1489–1499. doi: 10.1378/chest.12-1219 23648914 PMC3734884

[34] Ianella G, Maniaci A, Magliulo G et al. Current changes in the diagnosis and treatment of obstructive sleep apnea syndrome in the elderly. Pol Arch Intern Med 2020; 130: 649–654. doi: 10.20452/pamw.15283 32250579

[35] Beninati W, Harris CD, Herold DL, Shepard JW. The effect of snoring and obstructive sleep apnea on the sleep quality of bed partners. Mayo Clin Proc. 1999; 74: 955–958. doi: 10.4065/74.10.955 10918859

[36] Wiggins CL, Schmidt-Nowara WW, Coultas DB, Samet JM. Comparison of self- and spouse reports of snoring and other symptoms associated with sleep apnea syndrome. Sleep. 1990; 13: 245–252. doi: 10.1093/sleep/13.3.245 2356396

