# Supplementary material for: Family aggregation of sleep characteristics: Results of the Heinz Nixdorf Recall and the Multi-Generation Study
Source: PLoS One. 2021 Jun 4;16(6):e0252828. doi: 10.1371/journal.pone.0252828 (PMC8177478; doi:10.1371/journal.pone.0252828)
Supplement: S1 Table — (DOCX) [file pone.0252828.s001.docx]

**S1 Table.** Additional characteristics of index persons and their partners: the Heinz Nixdorf Recall (HNR) and the MultiGenerationStudy (MGS) (analysis population A)

|  |  | | **Index person**  **(HNR T2)** | **Partners**  **(MGS)** | |
| --- | --- | --- | --- | --- | --- |
| N |  | | 1181 | 1181 | |
| Occupational status | Employed | | 286 (24.2%) | 305 (25.8%) | |
|  | unemployed | | 32 (2.7%) | 11 (0.9%) | |
|  | Pensioner | | 772 (65.4%) | 766 (64.9%) | |
|  | Inactive / housewife | | 72 (6.1%) | 90 (7.6%) | |
|  | Missing | | 19 (1.6%) | 9 (0.8%) | |
| Smoking | Current smoker | | 133 (11.3%) | 155 (13.1%) | |
|  | Former smoker | | 561 (47.5%) | 495 (41.9%) | |
|  | Never smoker | | 485 (41.1%) | 525 (44.5%) | |
|  | Missing | | 2 (0.2%) | 6 (0.5%) | |
| BMI (kg/m^2^) |  | | 28.0 ± 4.8 | 27.9 ± 4.6 | |
| Hypertension | Normal | | 400 (33.9%) | 352 (29.8%) | |
|  | prehypertension | | 485 (41.1%) | 518 (43.9%) | |
|  | stage 1 | | 243 (20.6%) | 247 (20.9%) | |
|  | stage 2 | | 52 (4.4%) | 59 (5.0%) | |
|  | Missing | | 1 (0.1%) | 5 (0.4%) | |
| Diabetes mellitus | Yes | | 150 (12.7%) | 144 (12.2%) | |
|  | No | | 1028 (87.0%) | 1031 (87.3%) | |
|  | Don´t know / missing | | 3 (0.3%) | 6 (0.5%) | |
| Cancer ever ^a^ | Yes | | 210 (17.8%) | 141 (11.9%) | |
|  | No | | 969 (82.0%) | 1033 (87.5%) | |
|  | Don´t know / missing | | 2 (0.2%) | 7 (0.6%) | |
| Stroke ^b^ | Yes | | 38 (3.2%) | 51 (4.3%) | |
|  | No | | 1143 (96.8%) | 1124 (95.2%) | |
|  | Don´t know / missing | | 0 (0.0%) | 6 (0.5%) | |
| Coronary heart disease | Yes | | 117 (9.9%) | 103 (8.7%) | |
|  | No | | 1064 (90.1%) | 1072 (90.8%) | |
|  | Don´t know / missing | | 0 (0.0%) | 6 (0.5%) | |
| Antihypertensive drugs | Yes | | 635 (53.8%) | 600 (50.8%) | |
|  | No | | 546 (46.2%) | 581 (49.2%) | |
| Beta blockers | Yes | | 374 (31.7%) | 341 (28.9%) | |
|  | No | | 807 (68.3%) | 840 (71.1%) | |
| Cholesterol lowering drugs | Yes | 342 (29.0%) | | | 318 (26.9%) |
|  | No | 839 (71.0%) | | | 863 (73.1%) |
| Benzodiazepines | Yes | 9 (0.8%) | | | 10 (0.9%) |
|  | no | 1172 (99.2%) | | | 1171 (99.1%) |

mean ± standard deviation; n (proportions (%))

HNR T2: third visit to the study center in the Heinz Nixdorf Recall Study; MGS: MultiGeneration Study

^a^ Index persons were asked whether they had ever had cancer whereas partners were asked whether they had ever had cancer except skin cancer.

^b^ Index persons were asked whether they had ever had stroke whereas partners were asked whether they had ever had stroke or a transient ischemic attack.
