# Supplementary material for: Family aggregation of sleep characteristics: Results of the Heinz Nixdorf Recall and the Multi-Generation Study
Source: PLoS One. 2021 Jun 4;16(6):e0252828. doi: 10.1371/journal.pone.0252828 (PMC8177478; doi:10.1371/journal.pone.0252828)
Supplement: S2 Table — (DOCX) [file pone.0252828.s002.docx]

**S2 Table.** Additional characteristics of index persons and their children: the Heinz Nixdorf Recall (HNR) and the MultiGenerationStudy (MGS) (analysis population B)

|  |  | **Index person**  **per family**  **(HNR T2)** | **Index person**  **per child**  **(HNR T2)** | **Children**  **(MGS)** |
| --- | --- | --- | --- | --- |
| N |  | 1083 | 1497 | 1497 |
| Occupational status | employed | 223 (20.6%) | 312 (20.8%) | 1302 (87.0%) |
|  | unemployed | 30 (2.8%) | 40 (2.7%) | 31 (2.1%) |
|  | pensioner | 744 (68.7%) | 1023 (68.3%) | 26 (1.7%) |
|  | Inactive / housewife | 61 (5.6%) | 84 (5.6%) | 128 (8.6%) |
|  | missing | 25 (2.3%) | 38 (2.5%) | 10 (0.7%) |
| Smoking | Current smoker | 112 (10.3%) | 158 (10.6%) | 366 (24.4%) |
|  | Former smoker | 472 (43.6%) | 645 (43.1%) | 397 (26.5%) |
|  | Never smoker | 497 (45.9%) | 689 (46.0%) | 733 (49.0%) |
|  | Missing | 2 (0.2%) | 5 (0.3%) | 1 (0.1%) |
| BMI (kg/m^2^) |  | 28.5 ± 4.7 | 28.5 ± 4.8 | 25.8 ± 5.0 |
| Hypertension | normal | 353 (32.6%) | 494 (33.0%) | 667 (44.6%) |
|  | prehypertension | 430 (39.7%) | 589 (39.3%) | 649 (43.4%) |
|  | stage 1 | 235 (21.7%) | 328 (21.9%) | 157 (10.5%) |
|  | stage 2 | 63 (5.8%) | 82 (5.5%) | 20 (1.3%) |
|  | missing | 2 (0.2%) | 4 (0.3%) | 4 (0.3%) |
| Diabetes mellitus | Yes | 129 (11.9%) | 174 (11.7%) | 47 (3.1%) |
|  | No | 9949 (87.9%) | 1314 (87.8%) | 1447 (96.7%) |
|  | Don´t know/ missing | 5 (0.5%) | 9 (0.6%) | 3 (0.2%) |
| Cancer ever ^a^ | Yes | 187 (17.3%) | 256 (17.1%) | 34 (2.3%) |
|  | No | 891 (82.3%) | 1232 (82.3%) | 1460 (97.5%) |
|  | Don´t know/ missing | 5 (0.5%) | 9 (0.6%) | 3 (0.2%) |
| Stroke ^b^ | Yes | 35 (3.2%) | 49 (3.3%) | 12 (0.8%) |
|  | No | 1048 (96.8%) | 1448 (96.7%) | 1480 (98.9%) |
|  | Don´t know/ missing | 0 | 0 | 5 (0.3%) |
| Coronary heart disease | Yes | 100 (9.2%) | 137 (9.2%) | 13 (0.9%) |
|  | No | 983 (90.8%) | 1360 (90.8%) | 1481 (98.9%) |
|  | Don´t know/ missing | 0 | 0 | 3 (0.2%) |
| Antihypertensive drugs | Yes | 603 (55.7%) | 817 (54.6%) | 167 (11.2%) |
|  | No | 480 (44.3%) | 680 (45.4%) | 1330 (88.8%) |
| Beta blockers | Yes | 375 (34.6%) | 507 (33.9%) | 94 (6.3%) |
|  | No | 708 (65.4%) | 990 (66.1%) | 1403 (93.7%) |
| Cholesterol lowering drugs | Yes | 307 (28.4%) | 421 (28.1%) | 59 (3.9%) |
|  | No | 776 (71.7%) | 1076 (71.9%) | 1438 (96.1%) |
| Benzodiazepines | Yes | 6 (0.6%) | 7 (0.5%) | 4 (0.3%) |
|  | no | 1077 (99.4%) | 1490 (99.5%) | 1493 (99.7%) |

mean ± standard deviation; n (proportions (%))

HNR T2: third visit to the study center in the Heinz Nixdorf Recall Study; MGS: MultiGeneration Study

^a^ Index persons were asked whether they had ever had cancer whereas children were asked whether they had ever had cancer except skin cancer.

^b^ Index persons were asked whether they had ever had stroke whereas children were asked whether they had ever had stroke or a transient ischemic attack.
