# Supplementary material for: Family aggregation of sleep characteristics: Results of the Heinz Nixdorf Recall and the Multi-Generation Study
Source: PLoS One. 2021 Jun 4;16(6):e0252828. doi: 10.1371/journal.pone.0252828 (PMC8177478; doi:10.1371/journal.pone.0252828)
Supplement: S3 Table — (DOCX) [file pone.0252828.s003.docx]

**S3 Table.** Associations between sleep characteristics of index persons and their children, stratified by the median of parental age

|  | **Parental age ≤ 69 years** | | | **Parental age > 69 years** | | |
| --- | --- | --- | --- | --- | --- | --- |
| **Sleep characteristics of index persons** | **n** | **Sleep outcome of children ^a^** | **RR (95% CI)** | **n** | **Sleep outcome of children ^a^** | **RR (95% CI)** |
| Sleep quality |  |  |  |  |  |  |
| poor / very poor | 131 | 51 (38.9%) | 1.48  (1.15 – 1.91) | 133 | 41 (30.8%) | 1.10  (0.82 – 1.46) |
| good / very good | 573 | 151 (26.4%) | 1 | 572 | 161 (28.1%) | 1 |
| Snoring |  |  |  |  |  |  |
| Yes | 294 | 112 (38.1%) | 1.52  (1.09 – 2.14) | 168 | 85 (50.6%) | 1.03  (0.84 – 1.26) |
| No (ref) | 124 | 31 (25.0%) | 1 | 203 | 100 (49.3%) | 1 |
| Napping |  |  |  |  |  |  |
| ≥ 1 time / week | 222 | 57 (25.7%) | 2.07  (1.49 – 2.87) | 384 | 74 (19.3%) | 1.21  (0.87 – 1.69) |
| < 1 time / week (ref) | 467 | 58 (12.4%) | 1 | 295 | 47 (15.9%) | 1 |
| Difficulties falling asleep |  |  |  |  |  |  |
| ≥ 3 times / week | 73 | 8 (11.0%) | 1.09  (0.54 – 2.22) | 82 | 7 (8.5%) | 1.23  (0.56 – 2.69) |
| < 3 times / week | 458 | 46 (10.0%) | 1 | 460 | 32 (7.0%) | 1 |
| Difficulties maintaining sleep / early morning awakening |  |  |  |  |  |  |
| ≥ 3 times / week | 121 | 29 (24.0%) | 1.20  (0.83 – 1.75) | 130 | 31 (23.9%) | 1.38  (0.95 – 2.01) |
| < 3 times / week | 407 | 81 (19.9%) | 1 | 411 | 71 (17.3%) | 1 |
| Preference for getting up early ^b^ |  |  |  |  |  |  |
| Yes | 21 | 2 (9.5%) | 2.70  (0.67 – 10.9) | 40 | 4 (10.0%) | 1.64  (0.61 – 4.42) |
| No | 511 | 18 (3.5%) | 1 | 508 | 31 (6.1%) | 1 |
| Preference for getting up late ^c^ |  |  |  |  |  |  |
| Yes | 135 | 63 (46.7%) | 1.53  (1.21 – 1.94) | 89 | 32 (36.0%) | 1.50  (1.09 – 2.07) |
| No | 397 | 121 (30.5%) | 1 | 459 | 110 (24.0%) | 1 |

RR: relative risk; CI: confidence interval

^a^ For sleep quality of the index person as the exposure, the corresponding outcome of the children is poor / very poor sleep quality;

for snoring (yes / no) of the index person as the exposure, the corresponding outcome of the children is snoring (yes), etc

^b^ before 7 o´clock a.m.

^c^ at 9 o´clock a.m. or later
