# Supplementary material for: Family aggregation of sleep characteristics: Results of the Heinz Nixdorf Recall and the Multi-Generation Study
Source: PLoS One. 2021 Jun 4;16(6):e0252828. doi: 10.1371/journal.pone.0252828 (PMC8177478; doi:10.1371/journal.pone.0252828)
Supplement: S4 Table — (DOCX) [file pone.0252828.s004.docx]

**S4 Table.** Crude and adjusted associations between sleep characteristics of index persons and their children

| **Sleep characteristics of index persons** | **n** | **Sleep outcome**  **of children ^a^** | | **RR (95% CI)**  **crude** | **RR (95% CI)**  **age sex adjusted** | **RR (95% CI)**  **fully adjusted ^b^** |
| --- | --- | --- | --- | --- | --- | --- |
| Sleep quality |  | |  |  |  |  |
| poor / very poor | 264 | | 92 (34.9%) | 1.28  (1.06 – 1.55) | 1.26  (1.04 – 1.53) | 1.27  (1.05 – 1.55) |
| good / very good | 1145 | | 312 (27.3%) | 1 | 1 | 1 |
| Snoring |  | |  |  |  |  |
| Yes | 462 | | 197 (42.6%) | 1.06  (0.90 – 1.26) | 1.17  (0.99 – 1.39) | 1.17  (0.98 – 1.39) |
| No (ref) | 327 | | 131 (40.1%) | 1 | 1 | 1 |
| Napping |  | |  |  |  |  |
| ≥ 1 time / week | 606 | | 131 (21.6%) | 1.57  (1.24 – 1.98) | 1.60  (1.26 – 2.04) | 1.64  (1.29 – 2.11) |
| < 1 time / week (ref) | 762 | | 105 (13.8%) | 1 | 1 | 1 |
| Difficulties falling asleep |  | |  |  |  |  |
| ≥ 3 times / week | 155 | | 15 (9.7%) | 1.14  (0.67 – 1.93) | 1.17  (0.68 – 1.99) | 1.21  (0.71 – 2.08) |
| < 3 times / week | 918 | | 78 (8.5%) | 1 | 1 | 1 |
| Difficulties maintaining sleep / early morning awakening |  | |  |  |  |  |
| ≥ 3 times / week | 251 | | 60 (23.9%) | 1.28  (0.99 – 1.67) | 1.27  (0.98 – 1.66) | 1.28  (0.98 – 1.67) |
| < 3 times / week | 818 | | 152 (18.6%) | 1 | 1 | 1 |
| Preference for getting up early ^c^ |  | |  |  |  |  |
| Yes | 61 | | 6 (9.8%) | 2.05  (0.91 – 4.59) | 2.00  (0.89 – 4.51) | 2.21  (0.98 – 4.97) |
| No | 1019 | | 49 (4.8%) | 1 | 1 | 1 |
| Preference for getting up late ^d^ |  | |  |  |  |  |
| Yes | 224 | | 95 (42.4%) | 1.57  (1.30 – 1.90) | 1.50  (1.24 – 1.81) | 1.42  (1.17 – 1.73) |
| No | 856 | | 231 (27.0%) | 1 | 1 | 1 |

RR: relative risk; CI: confidence interval

^a^ For sleep quality of the index person as the exposure, the corresponding outcome of the children is poor / very poor sleep quality;

for snoring (yes / no) of the index person as the exposure, the corresponding outcome of the children is snoring (yes), etc

^b^ adjusted for age, sex, BMI, smoking, chronic diseases (cancer, diabetes, stroke, coronary heart disease), and drug intake (antihypertensive

drugs, cholesterol lowering drugs, benzodiazepines)

^c^ before 7 o´clock a.m.

^d^ at 9 o´clock a.m. or later
